# Identifying hotspots of woody plant diversity and their relevance with home ranges of the critically endangered gibbon (*Nomascus hainanus*) across forest landscapes within a tropical nature reserve

**DOI:** 10.3389/fpls.2023.1283037

**Published:** 2023-12-01

**Authors:** Xinran Li, Zhidong Zhang, Wenxing Long, Runguo Zang

**Affiliations:** ^1^ Key Laboratory of Biodiversity Conservation of National Forestry and Grassland Administration, Ecology and Nature Conservation Institute, Chinese Academy of Forestry, Beijing, China; ^2^ Hebei Provincial Key Laboratory of Forest Trees Germplasm Resources and Forest Protection, College of Forestry, Agricultural University of Hebei, Baoding, China; ^3^ Wuzhishan National Long-Term Forest Ecosystem Monitoring Research Station, Hainan Key Laboratory for Sustainable Utilization of Tropical Bioresource, College of Forestry, Hainan University, Haikou, China; ^4^ Institute of Hainan National Park, Haikou, China; ^5^ Co-Innovation Center for Sustainable Forestry in Southern China, Nanjing Forestry University, Nanjing, China

**Keywords:** tropical rainforest, plant diversity pattern, hotspots, Hainan gibbon, species conservation, anthropogenic disturbance

## Abstract

**Introduction:**

To achieve effective conservation objectives, it is crucial to map biodiversity patterns and hotspots while considering multiple influencing factors. However, focusing solely on biodiversity hotspots is inadequate for species conservation on a landscape scale. This emphasizes the importance of integrating hotspots with the home ranges of species to identify priority conservation areas.

**Methods:**

Compiling the vegetation data with environmental and anthropogenic disturbance data collected from kilometer-grid plots in Bawangling Nature Reserve, Hainan, China, we analyzed the spatial distribution of plant diversity (species richness and Shannon-Wiener index), as well as the main drivers affecting these patterns. We also investigated the spatial distribution of hotspots using a threshold approach and compared them with the home ranges of the flagship species, Hainan gibbon (*Nomascus hainanus*).

**Result:**

Climate and soil are predominant drivers shaping the spatial pattern of plant diversity in Bawangling Nature Reserve, surpassing the influence of anthropogenic disturbance and topographic factors. Both diversity indices exhibit a generally similar pattern with exceptions in surrounding areas of Futouling and Elongling. The hotspots identified by the Shannon-Wiener index showed a higher spatial overlap with the home ranges of Hainan gibbon compared to the species richness hotspots. The recently established Hainan gibbon Group E in 2019, located 8 km away from the original Futouling habitat, does not coincide with identified hotspots.

**Discussion:**

Our findings indicate that the hotspots of plant diversity within the habitat of Hainan gibbon Group E are relatively limited, emphasizing the necessity of giving precedence to its conservation. Integrating hotspots with the home ranges of critically endangered species offers decision-makers valuable information to establish rational conservation networks in the context of changing environments, as well as a reference for habitat restoration of species.

## 1 Introduction

Delineating and understanding the spatial distribution patterns of plant diversity pose significant challenge in biogeography and macroecology (Divíšek and Chytrý, 2018). The importance of the spatial distribution pattern of biodiversity has been increasingly recognized since the last century, with a number of studies being carried out, including mapping of plant diversity and collating regional species numbers across ecoregions (Kier et al., 2005; Večeřa et al., 2019). However, in recent decades, the loss of natural biodiversity due to anthropogenic-induced environmental and Land Use and Land Cover Change (LUCC) has become increasingly prevalent (Jaureguiberry et al., 2022). According to the significance of forest woody plants in nature and their role in maintaining ecosystem function and stability (Tilman et al., 2014), it is important to study plant diversity at the landscape scale to ensure the long-term sustainability of ecosystem (Nadrowski et al., 2010).

Insufficient spatial information on plant diversity presents a difficulty for accurately estimating the plant diversity distribution, further hindering effective species conservation planning (Vassallo et al., 2020). There is a requirement for predictive models to simulate spatial distribution patterns of plant diversity. In recent decades, advancements in statistical methods and the accessibility of environmental data sets have significantly advanced the utilization of predictive models for mapping spatial patterns of plant diversity at larger scales (Kier et al., 2005; Večeřa et al., 2019). However, most studies have focused on coarser resolutions, such as using compiled grid atlases (Liu et al., 2017) and regional plant inventories (Ulloa Ulloa et al., 2017), to delineate the formation mechanism and driving factors of the spatial pattern of plant diversity, particularly the impact of environmental factors on plant diversity across a wide range. Although there is significant research conducted at both global and regional levels, the insufficient precision in spatial data related to plant diversity distribution poses a challenge in offering precise guidance for conserving and managing biodiversity in nature reserves on a landscape scale.

Although patterns of plant diversity are proven to be well predicted by environmental and anthropogenic factors (Sabatini et al., 2022), understanding the drivers of plant diversity patterns remains one of the most fundamental issues in macroecology(Testolin et al., 2021). Several factors have been shown to influence plant diversity, particularly within forested landscapes including precipitation seasonality (Kwon et al., 2019), temperature seasonality (Qian, 2013), soil moisture levels (Härdtle et al., 2003), soil nutrients (Perroni-Ventura et al., 2006), altitude range (Ferrer-Castan and Vetaas, 2005), and multiple anthropogenic factors (Mcpherson et al., 2008). Each of these factors represents distinct aspects of environmental conditions. Climatic factors provide plants with the necessary water and energy for growth, influencing the spatial distribution of vegetation (Chauvier et al., 2021). Soil factors affect vegetation growth by providing essential nutrients and water (Chauvier et al., 2021). Topographic factors, such as slope and aspect, can directly impact soil moisture and nutrient storage, or indirectly affect plant diversity by interacting with climate and vegetation (Stein et al., 2014). Anthropogenic disturbance not only contributes to the degradation of tropical forests and the destruction of original habitats but also results in the reduction or extinction of local species, consequently impacting the plant diversity of forest communities (Vollstädt et al., 2017).

Protecting biodiversity at the landscape scale is a crucial concern for conservation biology (Tilker et al., 2020). The identification of biodiversity hotspots serves as an effective way to determine priority areas for conservation and accomplish conservation goals. Biodiversity hotspots encompass geographical areas harboring abundant plant diversity, rare or threatened species, and high levels of threat intensity (Reid, 1998). Hotspots can draw the attention of conservation managers and decision makers to areas that are both valuable and vulnerable (Bagstad et al., 2017), making them essential for biodiversity conservation and planning. Given the finite conservation resources available to policy makers and decision makers, the identification of hotspots facilitates their systematic regulation of priority conservation areas and rational allocation of finite conservation resources (Reyers et al., 2009).

Hainan gibbon (*Nomascus hainanus*), one of the four great apes worldwide, is classified as critically endangered by the IUCN (Geissmann and Bleisch, 2020). Originally distributed extensively across Hainan Island, its population range has considerably contracted due to anthropogenic hunting and habitat alterations (Stone, 2011). Presently, only five populations 35 individuals persist within forest patches confined to the Bawangling Nature Reserve (Chan et al., 2020). Habitats for Groups A, B, C, and D are primarily situated within the tropical montane rainforest, spanning altitudes of 800 to 1200 meters (near Futouling). Group E inhabits the tropical lowland rainforest, spanning altitudes of 400 to 800 meters (near Dongbengling). Revered as an umbrella species within nature reserves, the Hainan gibbon holds considerable ecological, economic, and societal values (Chan et al., 2005).

The integration of biodiversity hotspots with biological conservation at the landscape scale has emerged as a crucial concern for conservation biologists (Reid, 1998). Previous studies have demonstrated that integrating hotspots distribution with specifically chosen layers tailored to diverse research objectives, including anthropogenic disturbance factors (Doré et al., 2022) and networks of protected areas (Casanelles-Abella et al., 2023), holds the potential to provide valuable insights for advancing biodiversity conservation efforts. Consequently, there is a growing interest in developing a more comprehensive approach to assess the effectiveness of hotspots, yet there remains a gap in knowledge concerning hotspots in relation to the conservation of rare and endangered species (Rutledge et al., 2001; Zhao et al., 2016). The spatial patterns of plant species composition and diversity distribution within habitats can influence primate population size and distribution (Camaratta et al., 2017; Souza-Alves et al., 2021). On one hand, since food constitutes the most fundamental and crucial survival resource for animals, Hainan gibbon predominantly rely on plants for sustenance, and the adequacy of their food supply directly influences their survival, reproduction, and population dynamics (Wang et al., 2022). On the other hand, Hainan gibbon consistently inhabit trees throughout their lives. During nighttime, they exhibit a preference for trees with a larger diameter at breast height and higher heights as their night lodging plants (Tang and Jin, 2021). Additionally, they tend to choose locations abundant in these preferred night lodging plants as their night lodging areas. In light of this, the present study overlaid an analysis of plant diversity hotspots onto the home ranges of Hainan gibbon to assess the spatial distribution of plant diversity within their habitat.

In our research, we focused on forest woody plants within Hainan Bawangling Nature Reserve to discern the spatial pattern of plant diversity, identify hotspots and their influencing factors, and overlay these plant diversity hotspots onto the home ranges of Hainan gibbon. Our study aims to: (1) identify the factors that may influence the spatial patterns of plant diversity and assess their relative importance; (2) determine the spatial distribution patterns of plant diversity and hotspots; (3) provide insights for the conservation of Hainan gibbon with reference to the plant diversity hotspots. We hypothesized that (1) the predicted spatial pattern of plant diversity would resemble the distribution of known plant diversity in the nature reserve; (2) climate and soil factors would be the main drivers of the spatial pattern of plant diversity at the landscape scale; (3) an overlap exists between the home ranges of Hainan gibbon and plant diversity hotspots.

## 2 Materials and methods

### 2.1 Study area

**Figure 1:**
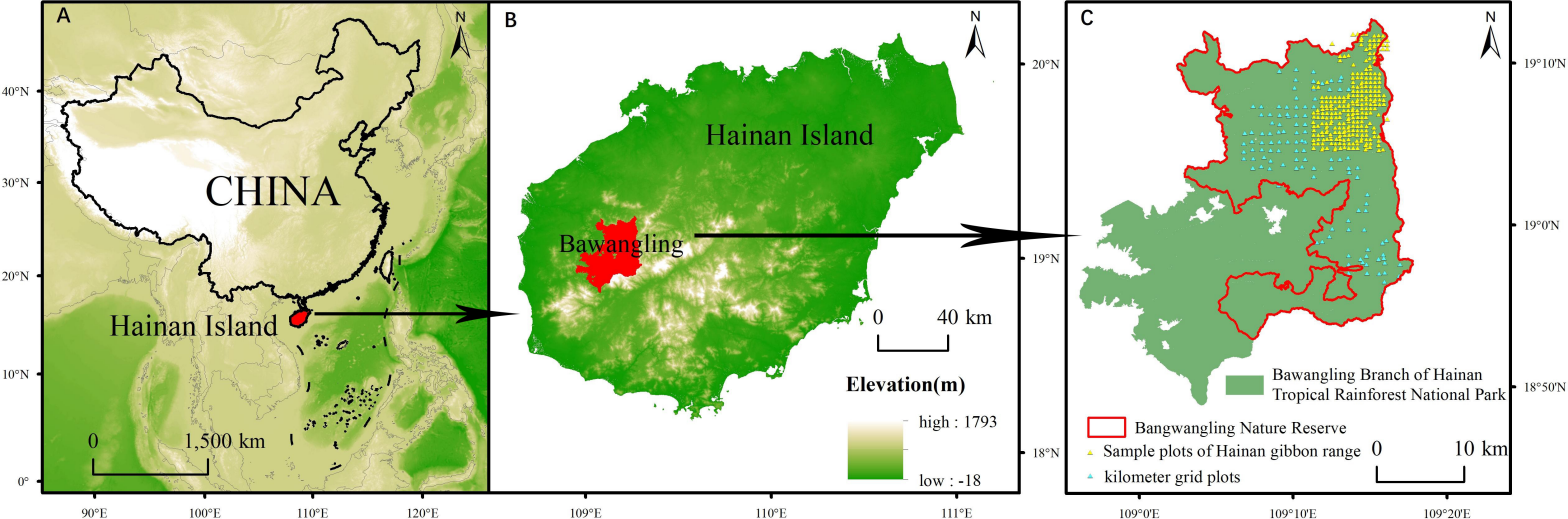
Location of Bawangling Nature Reserve in China. **(A)** Location of Hainan Island in China. **(B)** Location of Bawangling Branch of Hainan Tropical Rainforest National Park on Hainan Island. **(C)** Location of Bawangling Nature Reserve on Bawangling Branch of Hainan Tropical Rainforest.

Bawangling Nature Reserve (18°52’-19°12’ N, 108°53’-109°20’ E) is situated in the southwest of Hainan Province, China (**Figures 1A, B**). The reserve is primarily mountainous, with intricate terrain ranging from 123 to 1648 meters above sea level. It experiences an average annual precipitation of 1751 mm and an average annual temperature of 23.6°C;. The major vegetation types in the reserve include tropical lowland rainforest, tropical monsoon forest, tropical coniferous forest, tropical montane rainforest, tropical montane evergreen forest, and tropical hilltop dwarf forest. Over the past century, the primary anthropogenic disruptions to the wildlife and woody plants in the nature reserve have included tree clear-cutting, minority grazing, and poaching.

### 2.2 Woody plant species data

We established 391 community survey plots within the nature reserve, utilizing the grid as a reference (**Figure 1C**). Specifically, each plot was centered around a grid node measuring either 1 km × 1 km or 2 km × 1 km (totaling 114 plots), and grid nodes measuring 0.5 km × 0.5 km in the distribution area of Hainan gibbon served as additional centers (a total of 277 plots). Each 20m × 20m plot was further divided into four 10m × 10m subplots. The species name (specimens of unidentifiable species are collected and then identified), diameter at breast height (DBH), tree height, and crown width of trees and shrubs with a diameter ≥1cm in the plots were recorded, and the latitude and longitude coordinates of each plot were recorded with GPS in the center of the plot. From the community survey data, we calculated both species richness and the Shannon-Wiener index for each plot (**Table 1**).

**Table 1:** Descriptions of two plant diversity indices.

Diversity metric	Abbreviation& equation	Details	Characteristics
Species Richness	SR=S	S is the number of wood plant species within a plot	Widespread and common
Shannon-Wiener	SW= $\sum_{i=1}^{S}P_{i}lnP_{i}$	S is the number of species, Pi is the proportion of individuals belonging to species i among all individual species	Combining evenness or uniformity in the distribution of individuals in the species

### 2.3 Home ranges of Hainan gibbon

Through the aggregation of location coordinates from the monitoring records of Hainan gibbon’s activities within the Bawangling Branch of the Hainan Tropical Rainforest National Park Administration from 2000 to 2020, and integrating digitized topographic maps and satellite imageries of their habitat, we established the home ranges for Family Groups A, B, C, D, and E within the Bawangling Nature Reserve. Previous studies have indicated that the home ranges of Hainan gibbon spans approximately 1.49 km², with variations observed between wet and dry seasons (Bryant et al., 2017). In our analysis, we assumed a circular home ranges for Hainan gibbon and calculated a radius of 688 m based on the 1.49 km² area. To establish buffer zones around the primary home ranges, we applied a 688 m radius at the perimeter. These zones included the frequent home ranges (family group’s home ranges) and the infrequent home ranges (family group’s home ranges along with the buffer zone).

### 2.4 Predictor variables

We identified a set of candidate predictors encompassing the entire study area, with hypothesis that these factors influence the spatial distribution of plant species. These predictors were categorized into four groups: climate, soil, topography, and anthropogenic disturbance. Prior researches have been demonstrated their role in shaping plant diversity patterns (Ramírez-Marcial et al., 2001; Moeslund et al., 2013; Niskanen et al., 2017; Sabatini et al., 2022). To ensure consistency in the spatial resolution of environmental variables and the size of the measured sample sites (Niskanen et al., 2017), we resampled all environmental variables raster layers to a uniform image size of 20 m × 20 m across the entire study area (n = 1236954) using bilinear interpolation (Szymon and Radtke, 2017; Zhang et al., 2018). This approach ensured a unified resolution for prediction throughout the study. Then, the Pearson correlation coefficients among all explanatory variables were calculated and those with correlation coefficients greater than 0.85 were removed (**Figure S3**). Next, the variance inflation factor (VIF) was calculated, and select explanatory variables with VIF<10. Finally, we evaluated the importance of explanatory variables in the Random Forest model using the Increase in Mean Squared Error (%IncMSE). Beginning with the least significant variables, we sequentially removed variables until either the model accuracy was decreased or all relevant explanatory variables were excluded. We ultimately selected eleven variables to represent climate, soil, topography, and anthropogenic disturbance variables.

Nineteen climate factors incorporated into the model were obtained from the average annual climate data from 1970 to 2000 on the website of WorldClim (http://www.worldclim.org/), with a spatial resolution of 30 arc seconds. Soil variables were obtained from Soil SubCenter, National Earth System Science Data Center, National Science & Technology Infrastructure of China (http://soil.geodata.cn), with a horizontal spatial resolution of 90m (Liu et al., 2022). Nine soil variables were selected: pH, cation exchange capacity, bulk density, coarse fragments, total nitrogen, total phosphorus, total potassium, soil thickness, soil organic carbon. Topography variables came from the global digital elevation (DEM) model data released by National Aeronautics and Space Administration (NASA) in 2020, with a spatial resolution of 30m. Three topographic variables were used in this study: (1) elevation, representing the height of the terrain (2) slope, representing the degree of steepness of the surface unit (3) aspect, representing the direction of the ground’s slope.

We utilized a single anthropogenic disturbance factor generated by the Environmental Risk Surfaces model, with a spatial resolution of 20 m, which was derived from five risk elements: villages, factories, roads, artificial, and economic forests. The vector data of villages and roads were obtained from the 1: 250000 national basic geographic databases in the National Geographic Information Resources Catalog Service system. This data encompassed various road types, including national, provincial, county, township, and rural roads. Factory distribution points were extracted from Landsat remote sensing images through visual interpretation. Vector data of plantation and economic forest distribution were obtained from the Bawangling Forestry Bureau. The artificial forest mainly consisted of *Pinus caribaea*, *Pinus latteri*, and other tree species, while the economic forest was mainly composed of eucalyptus, rubber, Areca catechu, and other tree species. Disturbance intensity and distance of the various risk elements were determined based on the current situation of anthropogenic disturbance in the nature reserve and relevant literature (Mcpherson et al., 2008) (**Table S2**, **Figure S1**). The Environmental Risk Surfaces model incorporates four distance decay functions, namely linear, convex, concave, and constant, to represent the changes in interference intensity with distance. For this study, we adopted a linear function as the distance decay function. This function assumes a consistent rate of decrease in interference intensity as distance increases, until reaching the maximum distance where interference intensity becomes zero. For each risk factor, we calculate its interference intensity value based on the interference intensity and distance. Subsequently, we summed up the interference intensity values for all risk factors to obtain the overall interference intensity value (**Figure S2**).

### 2.5 Modeling plant diversity drivers, spatial pattern, and identifying hotspots

We utilized Random Forest model to analyze the drivers of the two plant diversity indices. Various regression methods, including multiple linear regression and generalized linear model, were preliminarily tested. Ultimately, the Random Forest model was chosen due to its capacity to process high-dimensional data and adopt multiple tree voting. Previous studies have established the efficacy of this method in predicting diversity (Szymon and Radtke, 2017; Mallinis et al., 2020).

In the first step of model construction, we utilized the *caret* package (Kuhn, 2008) to fit two plant diversity indices (a total of 391 observations) with eleven explanatory variables. The Random Forest model was configured with 1000 ntree and evaluated based on Root Mean Square Error (RMSE) criteria. To identify the factors influencing plant diversity spatial patterns, we employed the %IncMSE index in the Random Forest model and conducted variance partitioning analysis to assess the significance of variables both individual action and interactions. Explanatory variables with %IncMSE index of over 15% were considered crucial to the explained variance of the model. Meanwhile, to understand how the explanatory variables affected the predictive model results, we examined the reliance between the response variables and each explanatory variable in the optimal prediction model using univariate partial dependence plots. These plots presented the relationship between each explanatory variable and the Random Forest model predicted plant diversity, accounting for the effects of other variables considered, to identify the most desirable range of values for predicted plant diversity (Marini et al., 2015).

In the second step, we evaluated the model using appropriate metrics to identify the optimal prediction model. The explanatory variables of all 20m × 20m grid cells covering the nature reserve (n = 1236954) were used to fit predictive maps of the two plant diversity indices (Niskanen et al., 2017; Večeřa et al., 2019)., A bias-correction algorithm was applied to correct the values of the plant diversity indices. These steps enabled us to produce accurate prediction maps for the two plant diversity indices. To evaluate the accuracy of the prediction model, a ten-fold cross-validation method was used to estimate its precision. Model performance was assessed using the R^2^, Mean Absolute Error (MAE), and RMSE obtained through cross-validation (See **Figure S4** for more details). To optimize the prediction model, the random forest model included an adjustment parameter (mtry), which represents the number of variables used to construct the decision tree. For comparison purposes, only the three models with the lowest RMSE values were presented (**Figures S4A, C**). The Random Forest model with the lowest RMSE value was chosen as the final plant diversity prediction model. When mtry was set to 13 for species richness and 2 for Shannon-Wiener index, the model had the smallest RMSE value, the largest R^2^ value, and the smallest MAE value. Additionally, we utilized the residual map (Dogan and Dogan, 2006), which is another facet-based prediction uncertainty indicator, to evaluate the accuracy of the prediction model (See **Figure S5** for more details). Based on the numerical distribution, we classified the uncertainty of the prediction distribution into three levels, which are Strongly predictive, Moderately predictive, Less predictive (**Table S3**). The results indicated that most regions are Strongly predictive and few regions are Less predictive (**Figure S5**). Overall, based on studies of relevant model assessment metrics and the spatial distribution of uncertainty (Dogan and Dogan, 2006; Szymon and Radtke, 2017), our analysis suggested that plant diversity model predictions are sufficiently accurate. Although Random Forest model have been widely used in many studies, their results exhibited bias due to the unweighted averaging of regression tree ensembles, resulting in outcomes biased toward the sample mean. The model showed overestimation of the low diversity index and underestimation of the high diversity index (**Figures S4B, D**). To alleviate this issue, a bias-correction algorithm known as regression of observed on estimated values was applied (Sabatini et al., 2022). This method was applicable to integrated tree machine learning models, such as the Random Forest model (Huang et al., 2016) and boosted regression tree model (Belitz and Stackelberg, 2021). Firstly, a linear regression was fitted to the observed and predicted values:


(1) $$S_{fit}=aS_{obs}+b$$

$S_{obs}$ was the plant diversity data calculated from the observed plots, and $S_{fit}$ was the predicted data corresponding to the observed plots obtained using the Random Forest model. Secondly, a deviation correction formula was applied, and the final plant diversity was fitted using the following equation:


(2) $$S_{fit}^{bc}=\text{max}\left[\frac{S_{fit}-b}{a},0\right]$$

Values of $S_{fit}^{bc}$ less than 0 were removed. The Random Forest model, regression parameters a and b were used to predict the spatial distribution of plant diversity at the landscape scale.

In the third step, we utilized spatial grid of diversity indices to identify hotspots. Specifically, the hotspots were determined as the grid cells in the top 10% of plant diversity, which is a widely used threshold (Rodríguez et al., 2015; Schröter and Remme, 2016).

**Figure 2:**
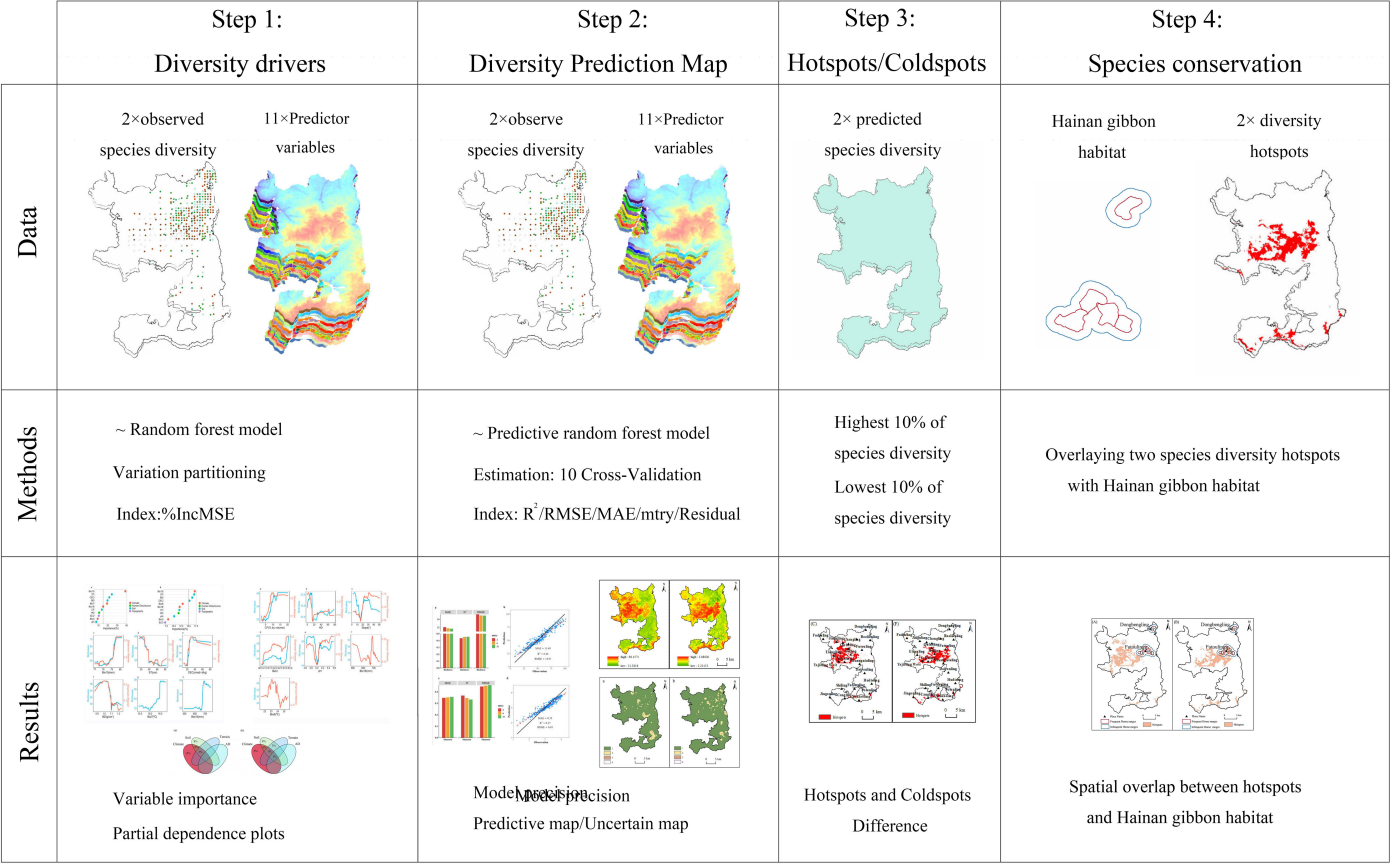
An overview of the data, methods, and results of a total of four stages from modeling to identify drivers and predict plant diversity to identifying hotspots and overlays.

Finally, to determine the consistency and difference between hotspots and the home ranges of Hainan gibbon, we overlaid the hotspots of species richness and Shannon-Wiener index diversity with the frequent and infrequent home ranges of Hainan gibbon, respectively. All data analysis and processing were performed using R version 4.2.2 (R Core Team, 2022) and ArcGIS version 10.8 (ESRI, 2020). **Figure 2** depicts the entire modeling and analysis process.

## 3 Results

### 3.1 Key drivers of the spatial distribution pattern of plant diversity

**Figure 3:**
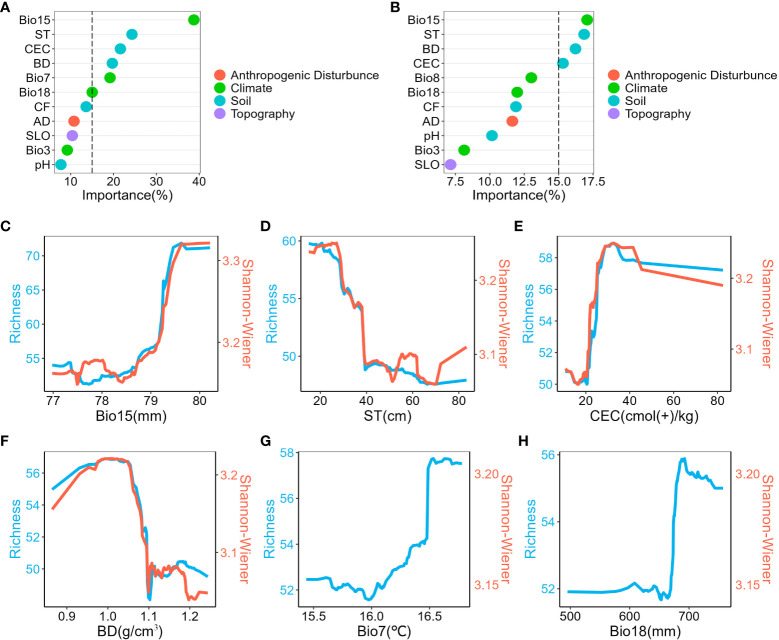
Relative Importance of predictors and univariate partial dependence plots. **(A)**, Relative importance of predictors in species richness model. **(B)**, Relative importance of predictors in the Shannon-Wiener index model. **(C–H)**, Univariate partial dependence plots are shown in descending order of importance of the variables in the species richness model. Show only variables with greater than 15% importance. Blue represents the species richness model and red represents the Shannon-Wiener index model. Bio15, precipitation seasonality; ST, soil thickness; CEC, cation exchange capacity; BD, bulk density; Bio7, temperature annual range; Bio18, precipitation of warmest quarter; Bio8, Mean Temperature of Wettest Quarter; CF, Coarse Fragments; AD, Anthropogenic Disturbance; SLO, Slope; Bio3, Isothermality.

**Figure 4:**
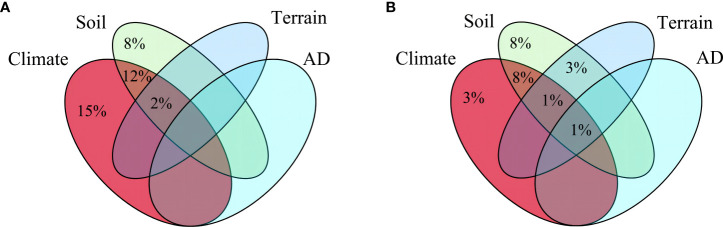
Results of variance partitioning of two plant diversity indices. **(A)**, Variation partitioning between species richness and explanatory variables. **(B)**, Variation partitioning between Shannon-Wiener index and explanatory variables. Only the fraction of explained proportions greater than 1% is shown. AD: anthropogenic disturbance.

Climate and soil factors acted as the main drivers of spatial diversity patterns, whereas anthropogenic disturbance and topography factors did not show significant impact. Regarding species richness, six variables were identified as important contributors: precipitation seasonality, soil thickness, cation exchange capacity, bulk density, temperature annual range, and precipitation of warmest quarter (**Figure 3A**). Conversely, the Shannon-Wiener index was influenced by four key variables: precipitation seasonality, soil thickness, bulk density, and cation exchange capacity (**Figure 3B**). Furthermore, the analysis of variance partitioning indicated that the combined effects of climate and soil were the most substantial factors, accounting for 12% of species richness and 8% of Shannon-Wiener Index, respectively (**Figure 4**).

Univariate partial dependence plots revealed consistent trends in the variables affecting plant diversity (**Figures 3C-H**, **Figure S6**). Specifically, as precipitation seasonality and coarse fragments increased, species richness and Shannon-Wiener index also increased significantly. In contrast, an increase in soil thickness was associated with a noticeable decrease in these indices. Nevertheless, the relationship between the remaining variables (bulk density, anthropogenic disturbance, slope, and pH) and plant diversity was non-linear, with fluctuating trends.

### 3.2 Spatial distribution pattern of plant diversity and hotspots

**Figure 5:**
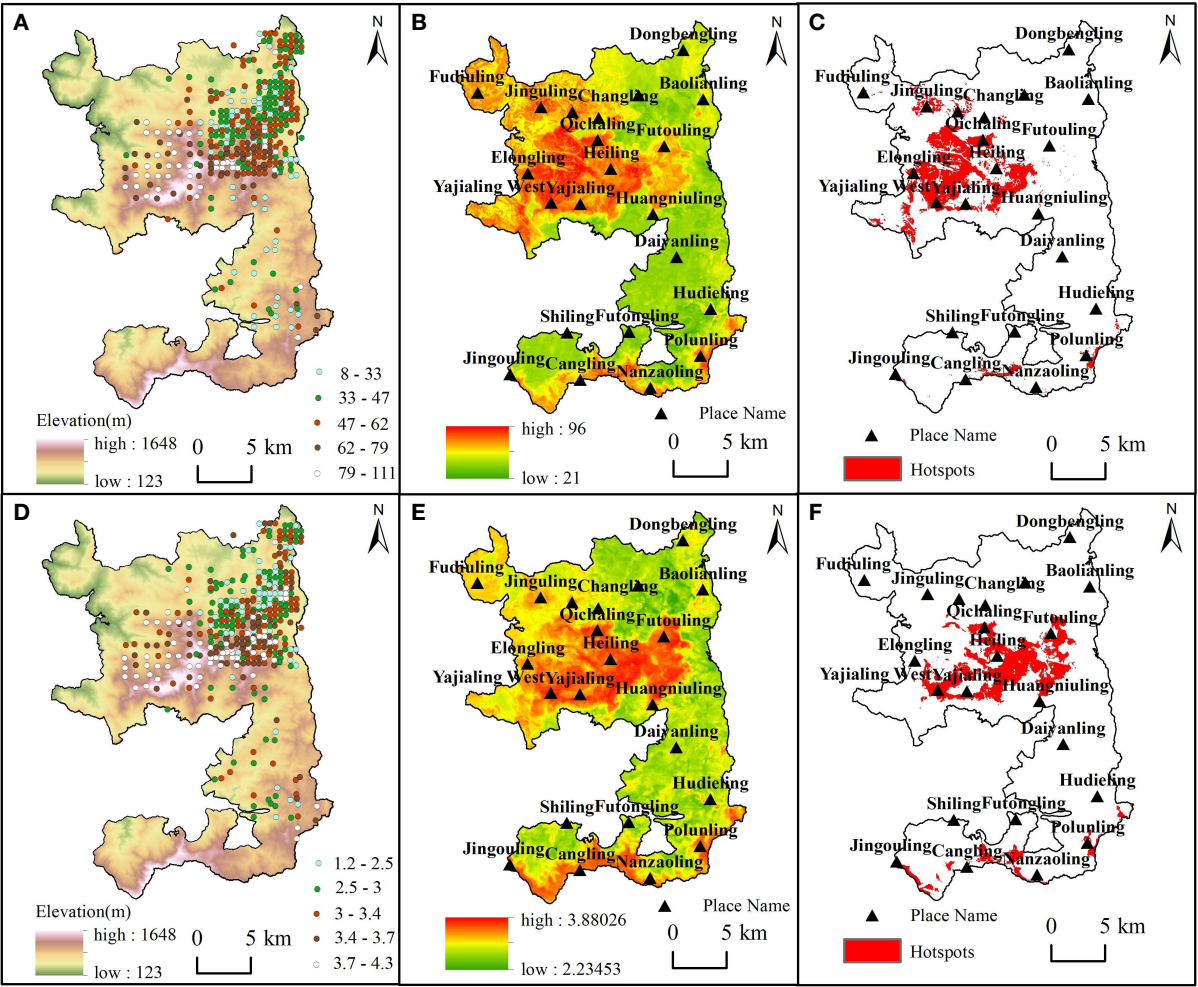
Spatial distribution patterns of plant diversity. **(A–C)**, Results on the spatial pattern of species richness. **(D–F)**, Results on the spatial pattern of Shannon-Wiener index. **(A, D)**, Spatial distribution of measured data. **(B, E)**, The spatial distribution map predicted by random forest model. **(C, F)**, The distribution of hotspots are based on the spatial distribution map.

The distribution of plant diversity hotspots varied, although there were similarities in the spatial patterns of species richness and Shannon-Wiener index (**Figure 5**). The best predictive model explained 46% and 37% of the variation in species richness and Shannon-Wiener index, respectively (**Figures S4B, D**). Areas with high species richness and Shannon-Wiener index were mainly concentrated in the central part of the nature reserve, while areas with low species richness and Shannon-Wiener index were located in the north of Futouling and south of Huangniuling (**Figures 5B, E**). Woody plant diversity hotspots in the forest landscape were relatively clustered and concentrated in the central area of the nature reserve, especially in Yajialing and Qichaling (**Figures 5C, F**). Notably, Futouling was encompassed by the hotspots of the Shannon-Wiener index but did not coincide with the hotspots of species richness. In contrast, Elongling was characterized by hotspots of species richness while lacking hotspots of the Shannon-Wiener index.

### 3.3 Conservation hotspots and priorities

**Figure 6:**
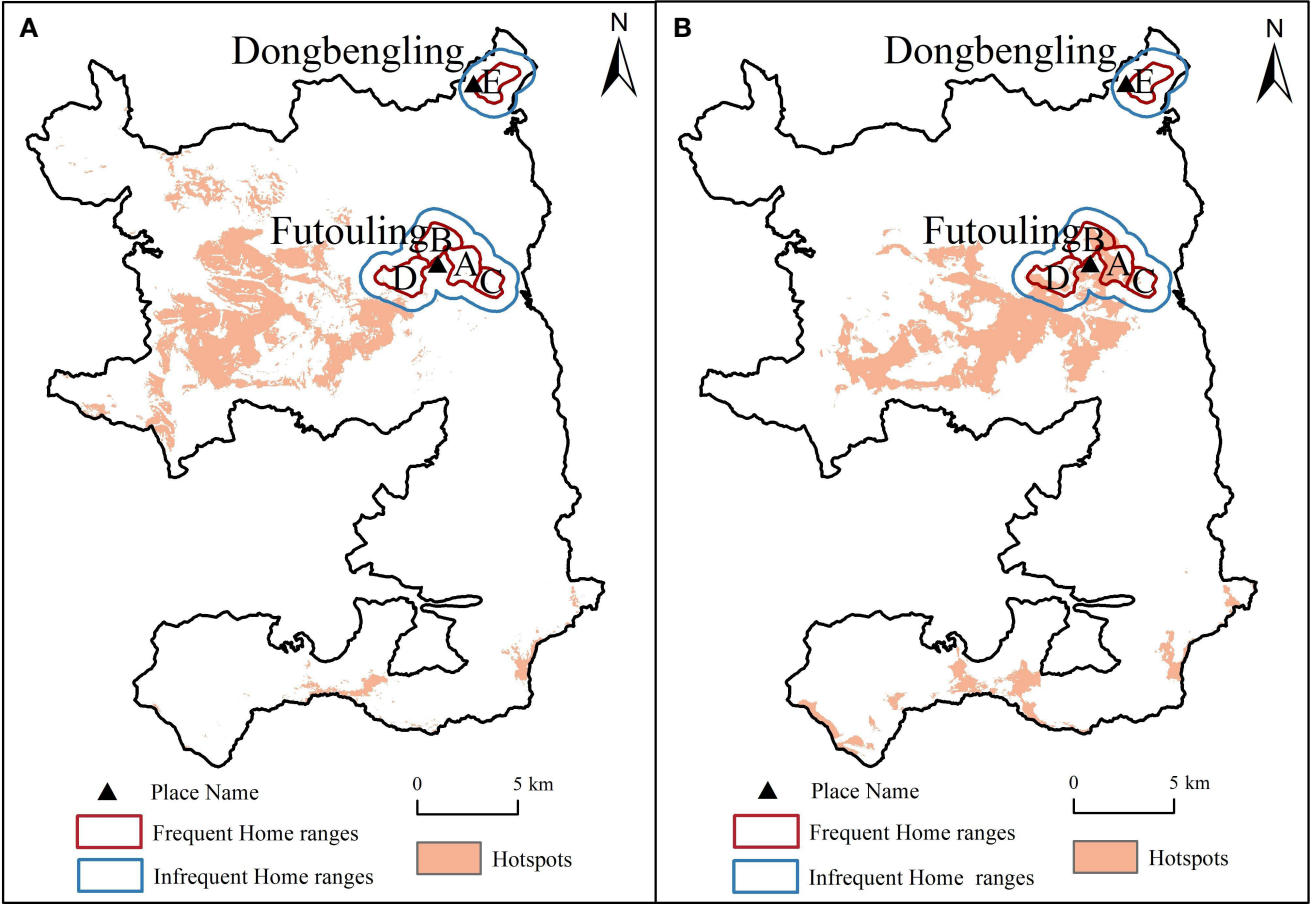
Relevance between hotspots and home ranges of Hainan gibbon. **(A)**, Species richness hotspots overlaid with home ranges of Hainan gibbon. **(B)**, Shannon-Wiener index hotspots overlaid with home ranges of Hainan gibbon.

The analysis of Hainan gibbon home ranges overlaid with plant diversity hotspots revealed that the gibbon had fewer overlaps with species richness hotspots, but more overlaps with Shannon-Wiener index hotspots (**Figure 6**). Furthermore, it is noteworthy that all four Hainan gibbon groups coincide with the plant diversity hotspots, except for Group E, which is situated in the upper right corner of the home ranges.

## 4 Discussion

### 4.1 Key driving factors of the spatial pattern of woody plant diversity in forest landscape

Our findings revealed that climate and soil factors were the main drivers of the spatial distribution pattern of plant diversity in the nature reserve (**Figures 3A, B**), which aligns with the hypothesis of this study. Precipitation seasonality, soil thickness, cation exchange capacity and bulk density had significant impacts on plant diversity indices. Numerous studies have indicated that the precipitation seasonality play a crucial role in determining plant diversity (Clinebell et al., 1995; Kwon et al., 2019). According to the water-energy dynamics hypothesis, variables associated with water and humidity are the main factors of plant diversity distribution patterns in tropical regions with favorable climatic conditions (Hawkins et al., 2003). Water, typically referring to liquid water, is directly linked to local precipitation levels. Higher levels of precipitation enhance water effectiveness within the soil, facilitating ion mobility, and ultimately promoting plant growth.

Bulk density and cation exchange capacity, to a certain extent, reflect soil fertility (Gustavo and Marco, 2013), a pivotal factor influencing various aspects of vegetation communities, including plant diversity (Huston, 1980), productivity (Wu et al., 2013), forest structure (Vitousek et al., 2010), and other characteristics of the forest ecosystem. Ecosystems with higher soil fertility offer more ecological niches and greater soil spatial heterogeneity due to species-specific physiological processes. Consequently, there is a decrease in nutrient competition among plants, which enhances the probability of species survival and contributes to higher plant diversity. Furthermore, the soil thickness significantly influences productivity (Romanyà and Vallejo, 2004), plant composition (Meyer et al., 2007), and plant diversity (Sun et al., 2017) through its impacts on water storage, nutrient uptake, water infiltration, and the size and distribution of plant roots. Thus, climate and soil factors are fundamental factors affecting the spatial pattern of plant diversity in the nature reserve, and our findings are consistent with previous studies(Stein et al., 2014; Kwon et al., 2019).

Anthropogenic disturbance factors were not identified as the main influencers of the plant diversity spatial distribution patterns. This could be attributed to the fact that the majority of the nature reserve consisted of natural forests. Additionally, compared to climate and soil factors, anthropogenic disturbance mainly indirectly impacts plant growth and diversity distribution through activities like selective logging and forest grazing. It does not play a significant main role in shaping spatial patterns of plant diversity at the landscape scale.

Regarding topographic factors, the selected variable (slope) exhibited limited explanatory power in explaining the spatial distribution of plant diversity. This suggests that the slope was not a critical driving factor for forest woody plant diversity in the nature reserve (**Figures 3A, B**). Previous studies have indicated that the topographic factors like elevation have a limited impact on plant diversity patterns when the elevation range remains relatively small and habitat heterogeneity appears to be comparatively low(Qian, 2013).

The unexplained variation in plant diversity may be attributed to local environmental conditions (Axmanová et al., 2012), forest management history (Horvat et al., 2017), and other unquantifiable factors. These factors which are difficult to quantify may include clear-cutting, selective cutting, and slash-and-burn cultivation, as well as local minority nomadic grazing in the last century before the establishment of the nature reserve, with management intensity and density varying widely in space and time. High-intensity regulatory practices (clear-cutting and slash-and-burn cultivation) have a dramatically negative effect on the plant diversity of forest woody plants (Paillet et al., 2010), while low-intensity forest management practices (selective logging and forest grazing) have a positive effect on plant diversity (Peringer et al., 2013). Although the effects of these factors on plant diversity were considered in this study, we were unable to fully quantify their impacts on plant diversity which could only be partially measured indirectly through changes in land cover type and anthropogenic disturbance factor.

### 4.2 Spatial pattern of woody plant diversity in forest landscape

The prediction model showed that predictors based on various environmental and anthropogenic disturbance factors explained 45% and 37% of the variation in plant species richness and Shannon-Wiener index, respectively. Similar methods have been used in other studies to simulate spatial patterns of plant diversity in forest communities. These studies explained 47% (Divíšek and Chytrý, 2018) and 55% (Lopatin et al., 2016) of the variation, but caution is needed when comparing predictions, as the amount of variation explained by plant diversity may be influenced by the study site and sample characteristics.

Our study provides information on the spatial distribution patterns of plant diversity in the nature reserve at landscape scales. The spatial distribution of species richness and Shannon-Wiener index observed in the predicted map closely matched the known natural vegetation composition in the nature reserve, supporting our hypothesis (**Figures 5A/D**). The central part of the reserve exhibits significantly higher species richness and Shannon-Wiener index than the upper and lower parts. This is primarily due to the distribution of two tropical rainforest types with the largest species richness and Shannon-Wiener index in the area, namely tropical lowland rainforest (700-900m above sea level) and tropical montane rainforest (700~1300m above sea level). These rainforests are distributed at higher altitudes, experience fewer anthropogenic disturbance, and possess more favorable climatic and soil conditions, leading to the higher plant diversity. However, the area to the northwest of Futouling and east of Fenshuiling has been affected by the nomadic pastoralism of ethnic minorities and anthropogenic activities in the last century. These activities, such as slash-and-burn cultivation, clear-cutting, and selective cutting, had persisted until the 1990s when logging of natural forests was gradually prohibited. Such activities led to severe damage to the forest community structure and had an extremely negative impact on plant diversity, resulting in the current existence of areas that are dominated by shrubs and plantation forests with low species richness and Shannon-Wiener index. The region extending from Daiyanling to the northern part of Hudieling contains Hainan Island’s largest and most densely natural tropical coniferous forest, dominated by *Pinus latteri*. This area receives lower precipitation levels compared to tropical lowland rainforest and tropical montane rainforest. Moreover, anthropogenic activities, such as resin cutting by Wangxia village residents, have had detrimental effects on plant diversity, resulting in a slight decrease in plant diversity.

### 4.3 Hotspots and their relevance with home ranges of Hainan gibbon

Overlay analyses for plant hotspots and distributions of endangered animals can help identify priority conservation areas. Kraft et al. (2010) contend that the overlap of floral neoendemism hotspots and the mammalian evolutionary hotspots in the central western region of California constitutes a vital region within the framework of the Conservation Plan. This plan serves a critical function in both the conservation and the generation of new biodiversity in California. Meanwhile, Hardouin and Hargreaves (2023) employed a comparative approach to assess hotspots within various taxonomic groups of nationally endangered species in Canada. The analysis revealed minimum hotspots overlap of only 16% between different endangered vascular plants and different endangered mammals. They identified high-risk hotspots, whether overlapping or not, as pivotal target areas for the expansion of protected zones and the facilitation of the recovery of endangered species. These studies demonstrate the effectiveness of conservation programs in hotspots-overlapping areas and underscore the urgent need for conservation measures in regions without such overlap.

The overlap between the hotspots of plant diversity indices and the home ranges of Hainan gibbon supports our third hypothesis. Our results showed differences not only in the overlap of the two diversity indices and the Hainan gibbon’s home ranges but also among various family groups of Hainan gibbon in hotspots regions. Compared to the Shannon-Wiener index hotspots, species richness hotspots rarely overlapped with the home ranges of Hainan gibbon (**Figure 6**). This result probably attributed to several factors. Firstly, it has been observed that areas with moderate anthropogenic disturbance tend to exhibit the highest levels of species richness (Connell, 1978). This suggests that regions experiencing a moderate level of human impact are more likely to support a diverse species. However, Hainan gibbon have specific habitat requirements for successful reproduction. Secondly, in areas with moderate disturbance, the structure of forest communities tends to be characterized by relatively simple secondary forests with a scarcity of large trees. Some studies (Fan et al., 2021; Zhang et al., 2022) highlight the significance of large trees for Hainan gibbon, as they serve as crucial sources of food and create a favorable environment for their survival. Unfortunately, the absence of a sufficient number of large trees and the relatively simplified forest structure pose challenges for Hainan gibbon in finding suitable resting and foraging conditions. As a result, Hainan gibbon is unable to effectively utilize these areas, hindering its ability to thrive and occupy species-rich regions.

An interesting observation from our study is that Group E did not coincide with two hotspots of plant diversity. This observation indicated that habitat of the newly formed Hainan gibbon Group E exhibits lower plant diversity compared to its original habitat. Group E separated from the original population around 2019 and currently occupies a habitat at an elevation of approximately 400-800m near Dongbengling, which is situated 8 km away from the original Futouling habitat (Chan et al., 2020; Zhang et al., 2022). The area is characterized by a combination of secondary forest, scrub grassland, and secondary lowland forest (Chan et al., 2020), potentially resulting in lower plant diversity compared to the original Futouling habitat. The presence of different forest types in their current location may be the primary reason for the lack of overlap between Group E and the hotspots. It is noteworthy that the suitable habitat for Hainan gibbon consist of tropical lowland rainforests below an elevation of 760 m (Zhang et al., 2021). However, the majority of the natural forests below 600 m on Hainan Island have been deforested to facilitate the expansion of monoculture plantations (Zhou et al., 2005). This situation has forced Hainan gibbon to migrate to tropical montane rainforests situated at elevations between 800 and 1200 m (Ren et al., 2022; Zhang et al., 2022). Although the plant diversity is lower near Dongbengling compared to the primary habitat near Futouling, it corresponds to a forest type and elevation preferred by Hainan gibbon. We thus predict that Hainan gibbon will continue to migrate to lower elevations as long as the plant diversity of the secondary forests at lower elevations meets their minimum survival requirements.

### 4.4 Conservation implication

Plant diversity hotspots play a crucial role in sustaining ecosystem services, emphasizing the need for increased attention and focus from conservation policy makers and decision makers. How can we implement protective measures for Hainan gibbon based on plant diversity hotspots? Firstly, it was noted that as the Hainan gibbon migrated from Futouling towards Dongbengling, and there was a lack of diversity hotspots in the habitat range between the two locations. Therefore, it is essential to create numerous ecological corridors linking Futouling and Dongbengling, enabling a connection between the habitats of Group E and Groups A, B, C and D. This will enhance the interconnectivity of suitable habitats for Hainan gibbon. Moreover, conservationists should prioritize enhancing plant diversity within the newly established home ranges of Group E. This can be achieved by planting the preferred vegetation of Hainan gibbon within the existing habitat of Group E, as a targeted conservation strategy for this endangered species. Enhancing plant diversity within its habitat can enhance the overall ecological health and resilience of the environment, benefiting not only the Hainan gibbon but also other species in the ecosystem.

In the future, biodiversity conservation planning and research should prioritize the use of various diversity indices (such as species diversity, functional diversity, and phylogenetic diversity) and multi-scale hotspots (global, regional, and local scales) (Crain et al., 2011) to identify priority areas for conservation. Moreover, considering the ongoing global warming trend, we suggest simulating future climate scenarios (SSP1-2.6, SSP2-4.5, and SSP5-8.5) as predictive variables. This will help forecast the spatial distribution and changing trends of biodiversity hotspots, enabling us to identify the spatial locations and changing circumstances of species shelters. As global warming continues, species are likely to migrate to higher altitudes (Le Roux and McGeoch, 2008), indicating that the spatial pattern of plant diversity will change with climate conditions. Hotspots are expected to shift towards higher altitudes in search of more suitable habitats for species. Plant diversity hotspots can serve as a unifying tool for aligning biodiversity conservation values among scientists, decision makers, and policy makers. This facilitatas the coordination of conservation concepts across various government departments, ultimately enabling the achievement of irreplaceable and long-term sustainability in biodiversity conservation within the nature reserve.

## 5 Conclusions

Overlaying the areas identified as plant diversity hotspots with the home ranges of Hainan gibbon can significantly enhance the balance between biodiversity assessment and the conservation of flagship species. Our findings demonstrated that the spatial distribution patterns of both plant diversity indices-based hotspots exhibit similarities yet display heterogeneity, predominantly driven by distinct environmental conditions. Remarkably, the recently established Group E of Hainan gibbon does not overlap with the hotspots. This observation suggests that the plant diversity within their habitat is relatively low, necessitating targeted policy interventions and meticulous management efforts to enhance the quality of their habitat. Furthermore, our discoveries aim to offer valuable insights for the conservation of flagship species. The spatial distribution of hotspots offers an invaluable reference for determining priority management levels, serving as a robust foundation for monitoring biodiversity across expansive landscapes and functioning as a crucial tool for biodiversity assessment and management.

## Data availability statement

The original contributions presented in the study are included in the article/**Supplementary Material**. Further inquiries can be directed to the corresponding author.

## Author contributions

XL: Formal analysis, Methodology, Writing – original draft. ZZ: Investigation, Writing – review & editing. WL: Investigation, Writing – review & editing. RZ: Writing – review & editing, Conceptualization.
