# Corrigendum: Identifying hotspots of woody plant diversity and their relevance with home ranges of the critically endangered gibbon *(Nomascus hainanus)* across forest landscapes within a tropical nature reserve

**DOI:** 10.3389/fpls.2024.1372724

**Published:** 2024-02-27

**Authors:** Xinran Li, Zhidong Zhang, Wenxing Long, Runguo Zang

**Affiliations:** ^1^ Key Laboratory of Biodiversity Conservation of National Forestry and Grassland Administration, Ecology and Nature Conservation Institute, Chinese Academy of Forestry, Beijing, China; ^2^ Hebei Provincial Key Laboratory of Forest Trees Germplasm Resources and Forest Protection, College of Forestry, Agricultural University of Hebei, Baoding, China; ^3^ Wuzhishan National Long-Term Forest Ecosystem Monitoring Research Station, Hainan Key Laboratory for Sustainable Utilization of Tropical Bioresource, College of Forestry, Hainan University, Haikou, China; ^4^ Institute of Hainan National Park, Haikou, China; ^5^ Co-Innovation Center for Sustainable Forestry in Southern China, Nanjing Forestry University, Nanjing, China

**Keywords:** tropical rainforest, plant diversity pattern, hotspots, Hainan gibbon, species conservation, anthropogenic disturbance

In the published article, there was an error in the Funding statement. The funding statement for the National Natural Science Foundation of China was displayed as “nU22A20503”. The correct statement is “the National Natural Science Foundation of China (U22A20503).’’. The correct Funding statement appears below.

**Funding**

The author(s) declare financial support was received for the research, authorship, and/or publication of this article. This research is supported by the National Natural Science Foundation of China (U22A20503).

The authors apologize for this error and state that this does not change the scientific conclusions of the article in any way. The original article has been updated.

