# Supplementary material for: Identifying hotspots of woody plant diversity and their relevance with home ranges of the critically endangered gibbon (Nomascus hainanus) across forest landscapes within a tropical nature reserve
Source: Front Plant Sci. 2023 Dec 1;14:1283037. doi: 10.3389/fpls.2023.1283037 (PMC10722271; doi:10.3389/fpls.2023.1283037)
Supplement: Supplementary file 1 [file DataSheet_1.docx]

**Supplementary Material**

**
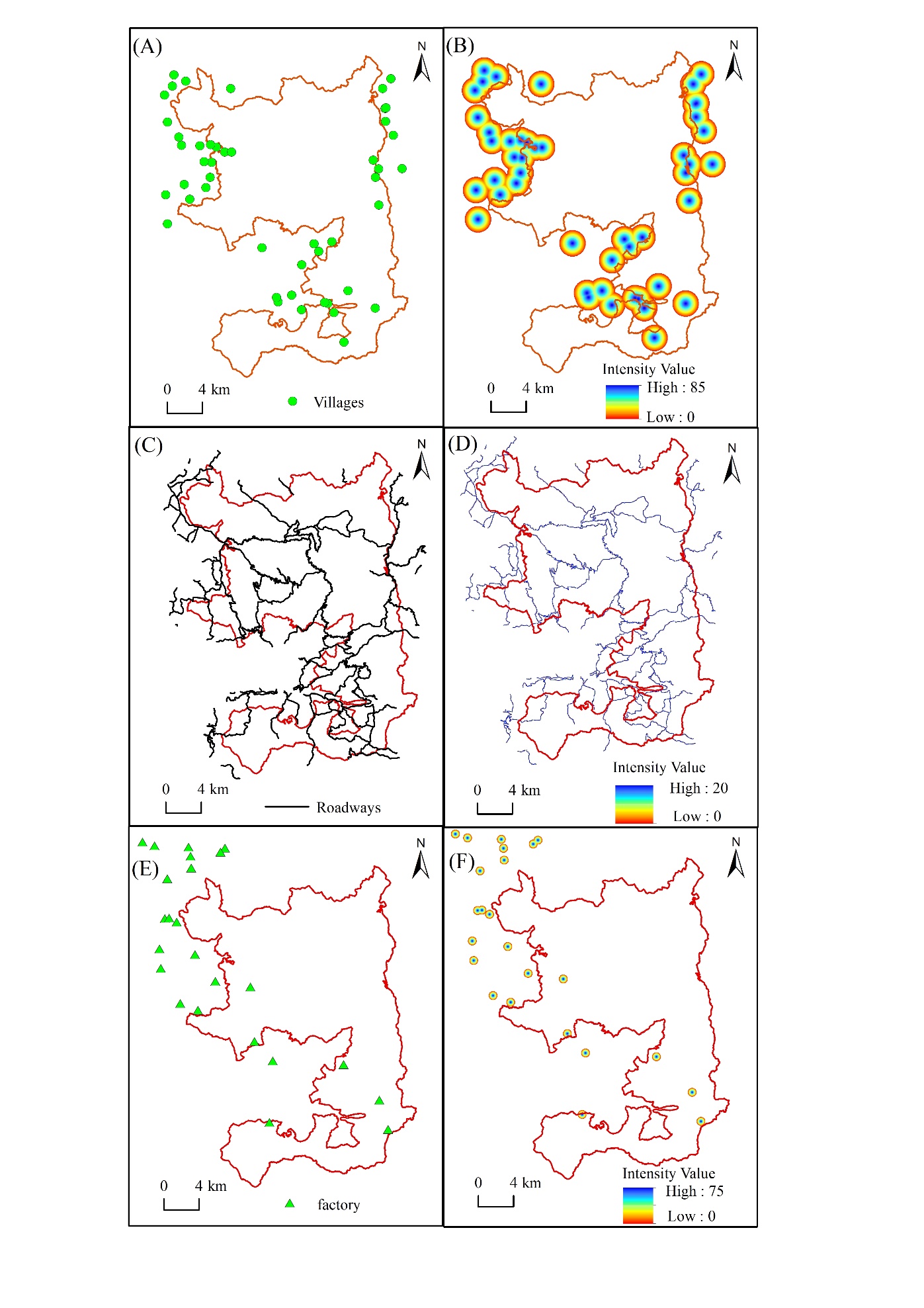
**

**
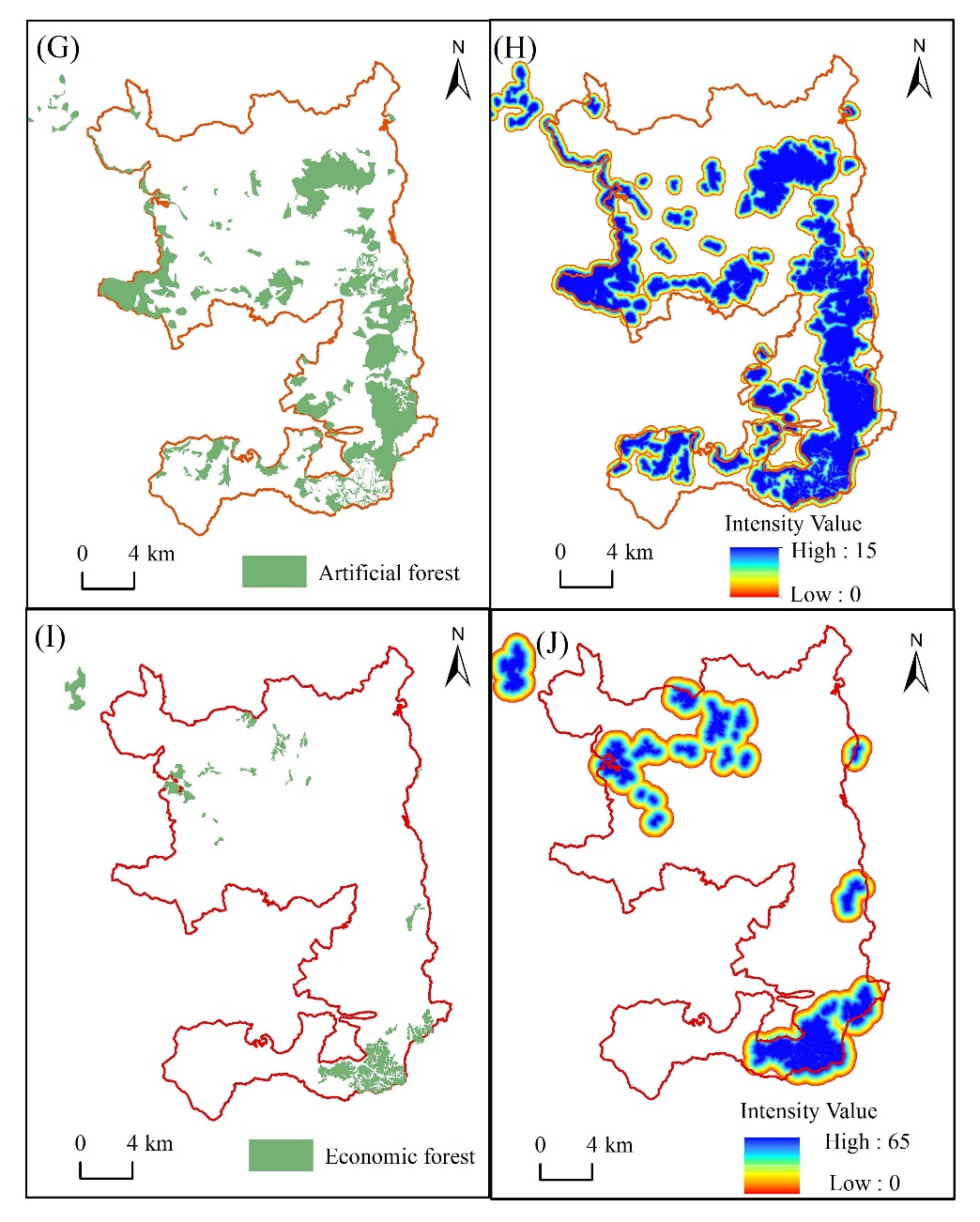
**

**FIGURE S1.** Spatial distribution and disturbance intensity distribution of dissimilar disturbance factors. (A-B), Village (C-D), Roadways (E-F), Factory (G-H), Artificial forest (I-J), Economic forest.


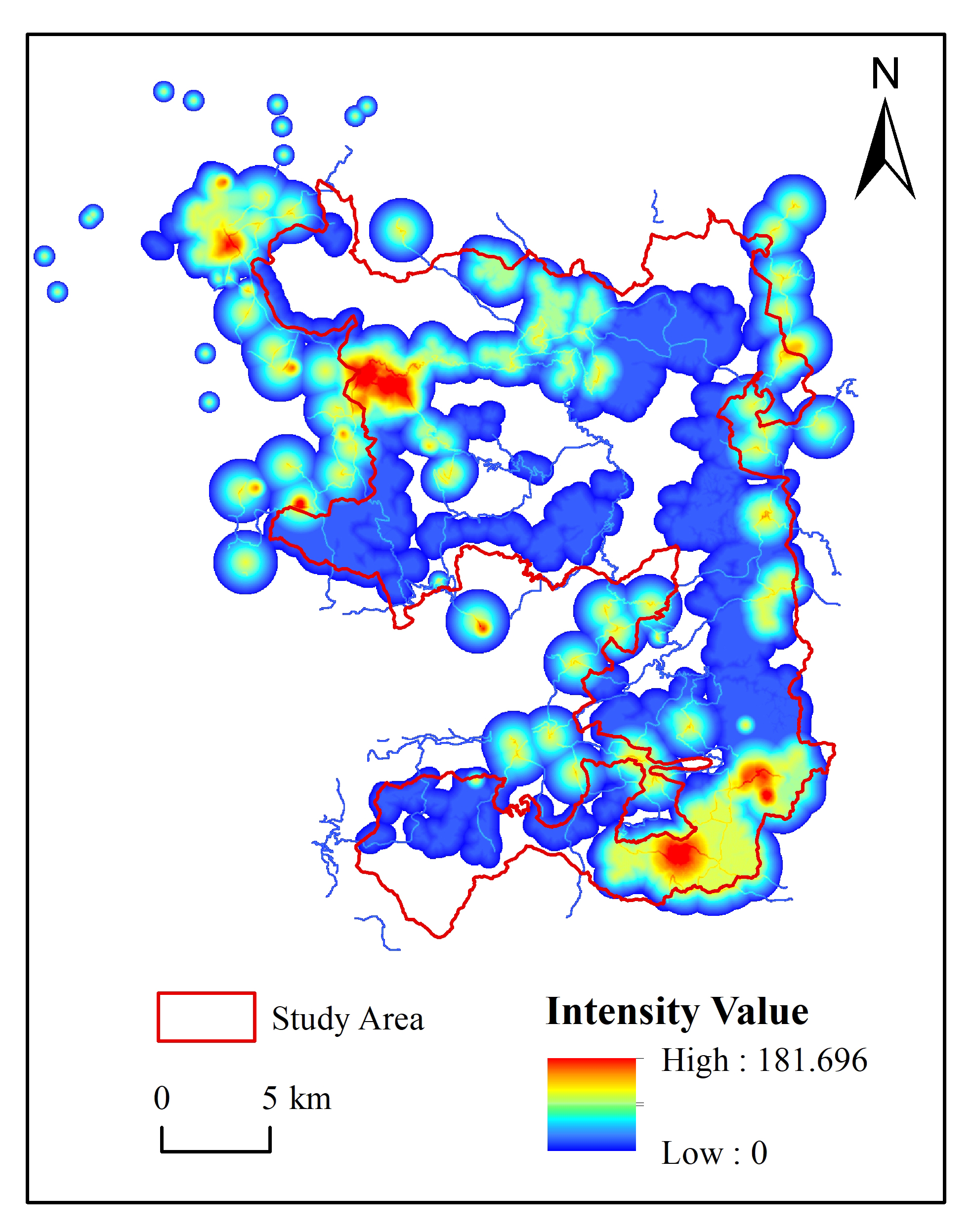


**FIGURE S2.** Synthetic diagram of anthropogenic disturbance intensity value.


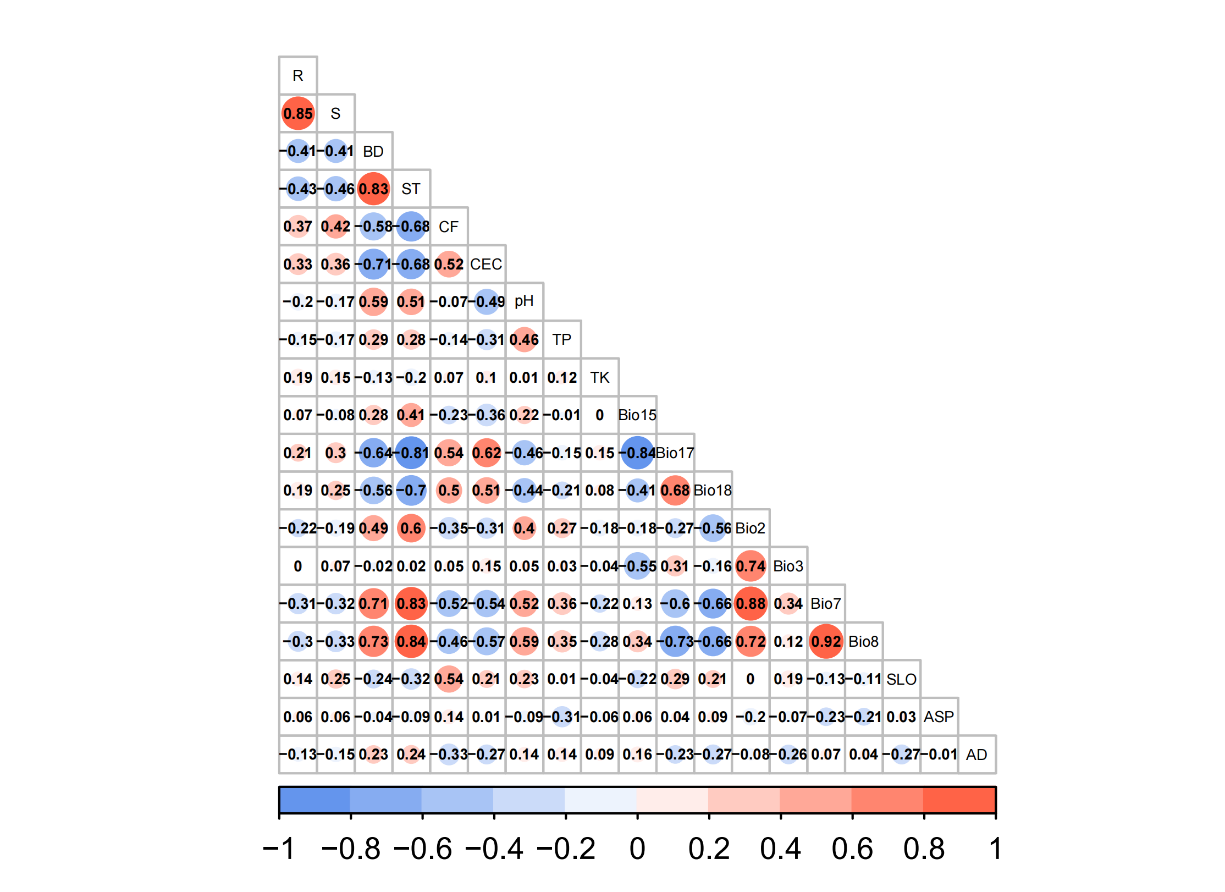


**FIGURE S3.** Pearson correlation coefficients between screened explanatory variables and plant diversity. R: richness, S: Shannon-Wiener index, Bio2: mean diurnal range, Bio3: isothermality, Bio7: temperature annual range, Bio8: mean temperature of wettest quarter, Bio15: precipitation seasonality, Bio17: precipitation of driest quarter, Bio18: precipitation of warmest quarter, ST: soil thickness, CF: coarse fragments, TK: total potassium, TP：total phosphorus, BD: bulk density, CEC: cation exchange capacity, SLO: slope, ASP: aspect, AD: anthropogenic disturbance.

**
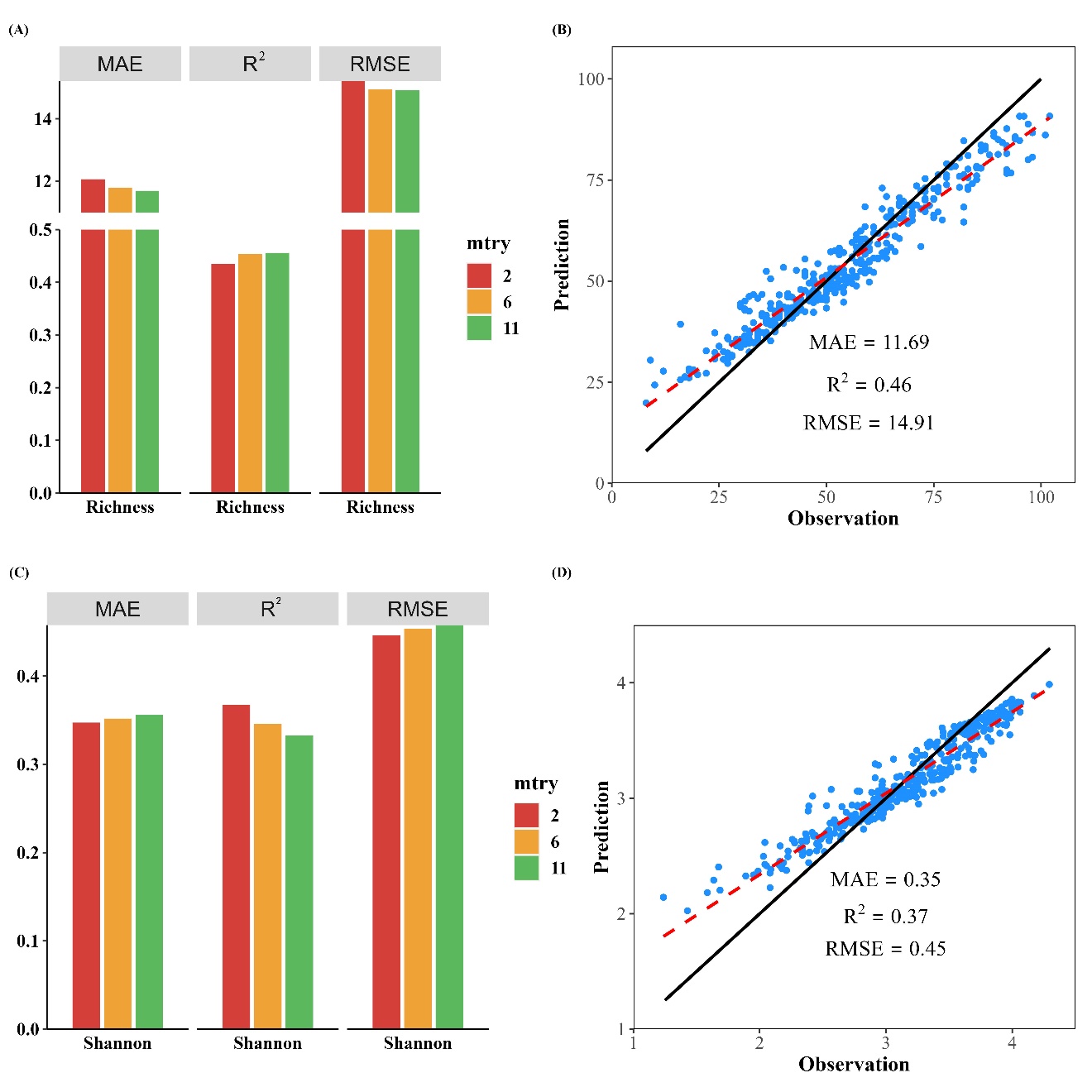
**

**FIGURE S4.** (A、C), The performance metrics of the prediction model (MAE: mean absolute error, R^2^, and RMSE: root mean square error) were compared for different mtry values. Among them, R^2^ was used to indicate the percentage of variation in the response variable that can be explained by the explanatory variable. A higher R^2^ value suggested that the model has a stronger explanatory power. RMSE, on the other hand, measures the degree of variation in the data points from the predicted values, with lower RMSE values indicating less deviation and higher accuracy. MAE was used to address the issue that RMSE can be greatly affected by outlier residuals. Lower MAE and RMSE values indicated a smaller deviation in the model and higher accuracy. (B、D), A scatter plot was generated to verify the accuracy of the RF model in estimating vegetation SR and SW in Bawangling Nature Reserve. The horizontal axis represents the measured sample data, while the vertical axis represents the predicted values based on the RF model. The red dotted line indicates the unitary linear regression of observations and predictions. The black line (slope equal to 1) represents the values where the observed value is equal to the predicted value.

**
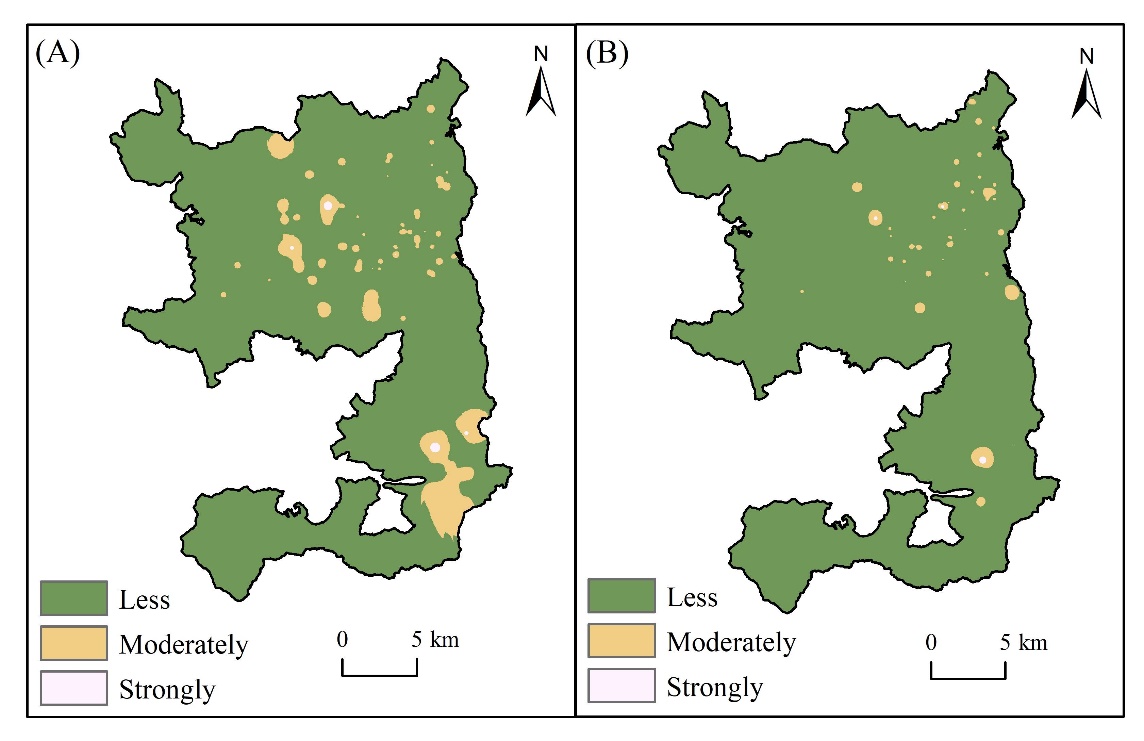
**

**FIGURE S5.** The residuals were obtained by calculating the absolute difference between the predicted and observed plant diversity values in each plot. We then interpolated the residual points into a residual distribution map using the Inverse Distance Weighted interpolation method and evaluated the uncertainty of the model prediction in the nature reserve. Less, moderately, and strongly represent less predictive(high residuals), moderately predictive (medium residuals), and strongly predictive(low residuals) respectively. (A), Uncertainty distribution map of the prediction model based on species richness. (B), Uncertainty distribution map of the prediction model based on Shannon-Wiener index.


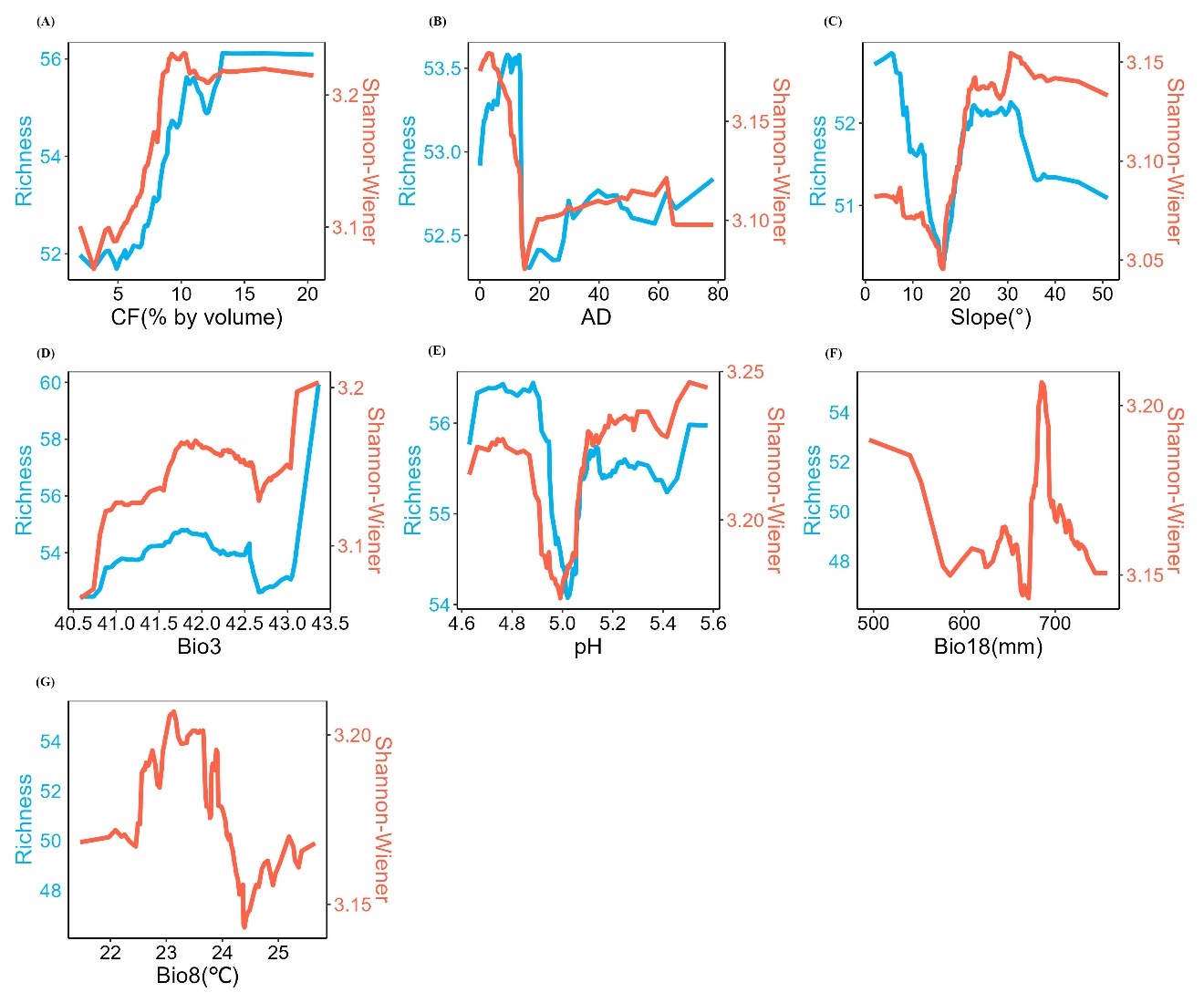


**FIGURE S6.** (A-G), Univariate partial dependence plots are shown in descending order of importance of the variables in the SR model. Blue represents the SR model and red represents the SW model. CF: coarse fragments, AD: anthropogenic disturbance, Bio3: Isothermality, Bio18: precipitation of warmest quarter, Bio8: mean temperature of wettest quarter.

**Table S1.**  Information about predictor variables

| Predictor variables | Abbreviation | Unit | Mean[min-max] | Source |
| --- | --- | --- | --- | --- |
| Annual Mean Temperature | Bio1 | ℃ | 21.2[17.8-24.6] | WorldClim |
| Mean Diurnal Range | Bio2 | ℃ | 7[6.4-7.6] | WorldClim |
| Isothermality | Bio3 | - | 42.2[40.5-43.9] | WorldClim |
| Temperature Seasonality | Bio4 | - | 342.45[316.9-368] | WorldClim |
| Max Temperature of Warmest Month | Bio5 | ℃ | 28.25[24.3-32.2] | WorldClim |
| Min Temperature of Warmest Month | Bio6 | ℃ | 12[9.1-14.9] | WorldClim |
| Temperature Annual Range | Bio7 | ℃ | 16.55[15.2-17.3] | WorldClim |
| Mean Temperature of Wettest Quarter | Bio8 | ℃ | 23.65[20.7-26.6] | WorldClim |
| Mean Temperature of Driest Quarter | Bio9 | ℃ | 16.6[13.5-19.7] | WorldClim |
| Mean Temperature of Warmest Quarter | Bio10 | ℃ | 24.75[21.1-28.4] | WorldClim |
| Mean Temperature of Coldest Quarter | Bio11 | ℃ | 33.2[13.5-19.7] | WorldClim |
| Annual Precipitation | Bio12 | mm | 1511.5[1283.7-1739.3] | WorldClim |
| Precipitation of Wettest Month | Bio13 | mm | 273.45[229-317.9] | WorldClim |
| Precipitation of Driest Month | Bio14 | mm | 15.5[10-21] | WorldClim |
| Precipitation Seasonality | Bio15 | mm | 78.75[76.8-80.7] | WorldClim |
| Precipitation of Wettest Quarter | Bio16 | mm | 762.6[644.6-880.6] | WorldClim |
| Precipitation of Driest Quarter | Bio17 | mm | 56.15[41.1-71.2] | WorldClim |
| Precipitation of Warmest Quarter | Bio18 | mm | 611.3[449-773.6] | WorldClim |
| Precipitation of Coldest Quarter | Bio19 | mm | 56.15[41.1-71.2] | WorldClim |
| Soil Thickness | ST | cm | 81.3[6.3-156.3] | National Earth System Science Data Center |
| Total Nitrogen | TN | g/Kg | 1.5 [0.2-2.8] | National Earth System Science Data Center |
| Total Potassium | TK | g/Kg | 14.7[0.9-28.4] | National Earth System Science Data Center |
| Total [Phosphorus](javascript:;) | TP | g/Kg | 0.8[0.09-1.6] | National Earth System Science Data Center |
| pH | pH | - | 5.5[4.3-6.6] | National Earth System Science Data Center |
| Cation Exchange Capacity | CEC | Cmol(+)/kg | 51.7[4.2-99.2] | National Earth System Science Data Center |
| Organic Carbon | SOC | g/Kg | 24.6[3.5-45.7] | National Earth System Science Data Center |
| Coarse Fragments | CF | % by volume | 14.5[0-29] | National Earth System Science Data Center |
| Bulk Density | BD | g/cm^3^ | 1.2[0.8-1.6] | National Earth System Science Data Center |
| Digital Elevation Model | DEM | - | 856.5[114-1599] | NASA |
| Aspect | AS | ° | 179.7[0-359.4] | NASA |
| Slope | SL | ° | 34.1[0-68.2] | NASA |
| Anthropogenic Disturbance | AD | - | 90.9[0-181.7] |  |

**Table S2.** Disturbance intensity and distance of different risk elements

| Risk elements | Disturbance intensity | Disturbance distance(m) |
| --- | --- | --- |
| Villages | 85 | 1500 |
| Roads | 20 | 60 |
| Factories | 75 | 500 |
| Artificial forest | 15 | 500 |
| Economic forest | 65 | 1000 |

**Table S3** Rating of residual uncertainty

| Residual absolute values of species richness | Residual absolute values of Shannon-Wiener index | Uncertainty level |
| --- | --- | --- |
| 0-8 | 0-0.3 | Strongly predictive |
| 8-16 | 0.3-0.6 | Moderately predictive |
| 16-24 | 0.6-0.9 | Less predictive |
